# Neurogenic Interventions for Fear Memory via Modulation of the Hippocampal Function and Neural Circuits

**DOI:** 10.3390/ijms23073582

**Published:** 2022-03-25

**Authors:** Hee Ra Park, Mudan Cai, Eun Jin Yang

**Affiliations:** Department of KM Science Research Division, Korea Institute of Oriental Medicine (KIOM), 1672 Yuseong-daero, Yuseong-gu, Daejeon 34054, Korea; hrpark0109@kiom.re.kr (H.R.P.); mudan126@kiom.re.kr (M.C.)

**Keywords:** fear memory, post-traumatic stress disorder, hippocampus, amygdala, medial prefrontal cortex, neural circuit

## Abstract

Fear memory helps animals and humans avoid harm from certain stimuli and coordinate adaptive behavior. However, excessive consolidation of fear memory, caused by the dysfunction of cellular mechanisms and neural circuits in the brain, is responsible for post-traumatic stress disorder and anxiety-related disorders. Dysregulation of specific brain regions and neural circuits, particularly the hippocampus, amygdala, and medial prefrontal cortex, have been demonstrated in patients with these disorders. These regions are involved in learning, memory, consolidation, and extinction. These are also the brain regions where new neurons are generated and are crucial for memory formation and integration. Therefore, these three brain regions and neural circuits have contributed greatly to studies on neural plasticity and structural remodeling in patients with psychiatric disorders. In this review, we provide an understanding of fear memory and its underlying cellular mechanisms and describe how neural circuits are involved in fear memory. Additionally, we discuss therapeutic interventions for these disorders based on their proneurogenic efficacy and the neural circuits involved in fear memory.

## 1. Introduction

Trauma is a physical or emotional response to a terrible event, such as an accident, natural disaster, medical trauma, war, terrorism, violence, forced separation, or witnessing a suicide. Trauma-induced fear is a normal reaction, resulting in temporary physiological and behavioral states that return to baseline after a period of time. However, excessive or repeated fear-based exposure to traumatic events can also lead to a pathological state of fear that underlies anxiety- or trauma-related disorders such as post-traumatic stress disorder (PTSD), which has a lifetime prevalence of approximately 3.6% according to a worldwide survey by the World Health Organization [1]. Fear is a conscious state consisting of both associative and nonassociative components, caused by exposure to real or imaginary threats. Healthy individuals who have experienced situations that share the characteristics of a traumatic event are able to discriminate traumatic memories from new experiences and successfully encode these experiences as new and safe. In contrast, individuals with PTSD or anxiety disorders may be unable to distinguish between their traumatic experience and new nontraumatic experiences due to their issues with extinction of fear-based traumatic memories, which leads to overgeneralization, excessive arousal, fear responses, and anxiety. Individuals with fear-based disorders often have impaired pattern separation, the process that minimizes overlaps between similar experiences and distributes them. These responses are closely related to the activities of brain regions and circuits, particularly the hippocampus, amygdala, and medial prefrontal cortex (mPFC). The pivotal functions of these brain areas are the formation, organization, and storage of new memories by comparing new sensory inputs to stored representations, as well as pattern separation. Moreover, they connect with existing neural circuits to integrate new memories and guide behavior. As fear memories and imprinting are the basis of PTSD and anxiety-related disorders, understanding the mechanisms underlying fear memories may help develop treatment options for these disorders. In this paper, we provide an overview of the hippocampus, its associated brain regions, and neural circuits for fear-based learning and memory. Additionally, we introduce new methodologies and therapeutic interventions for the treatment of fear-based disorders.

## 2. Methods

To conduct this review, we searched PubMed and Google for published papers that focused on fear memory and fear memory-related neural circuits as well as therapeutic interventions in preclinical and clinical studies on fear condition and perused them. The search keywords included fear memory, neural circuits, proneurogenic efficacy, therapeutic intervention, in vivo, and clinical study.

## 3. Fear Learning and Memory

Fear is one of the most basic human emotions triggered by perceived threats. It is a survival mechanism that sends signals to our bodies to initiate a fight-or-flight response in the face of danger. However, excessive or maladaptive fear learning and overgeneralization lead to the development of psychopathology. These are major features of anxiety and stress-related disorders, including PTSD, which can be viewed as a maladaptive fear response. Furthermore, fear learning causes powerful, long-lasting, and imprinted memories, as the acquisition of the memory (initial fear learning) is followed by a consolidation process in which the memory is thought to be stabilized.

In experiments on laboratory animals, the biological and physiological mechanisms of fear overgeneralization were evaluated using a fear conditioning paradigm [2,3]. Fear conditioning consists of the repeated pairing of a neutral stimulus, such as light or tone (conditioned stimulus (CS)), with an aversive stimulus, such as an electric footshock (unconditioned stimulus (US)). Presenting the CS alone after a few CS–US pairings can elicit measurable physiological arousal and response tendencies (e.g., freezing), mimicking the human conditioned fear response. This approach has provided insights into the neurobiological mechanisms involved in fear learning, memory, extinction, and overgeneralization.

## 4. Fear Memory and Overgeneralization in the Hippocampus

The hippocampus is located in the temporal lobe, with a longitudinal structure that can be functionally divided into the dorsal, intermediate, and ventral parts. Additionally, the hippocampus can be transversely divided into the CA1, CA3, and dentate gyrus (DG) regions. One of the pivotal functions of the hippocampus is the formation and reconstruction of memories by comparing new sensory inputs with stored representations, which, in turn, guides appropriate behaviors. The dorsal and ventral hippocampus contribute to both the consolidation of contextual and spatial memories and recall as well as fear memory generalization because the hippocampus receives afferent input from both the amygdala and the septum, which play important roles in fear memory. Interestingly, there is evidence that the subregions of the hippocampus have different effects on fear memory and extinction. Temporary inactivation of the dorsal hippocampus using lidocaine impaired avoidance behavior during retention, whereas inactivation of ventral hippocampus impaired fear memory acquisition [4,5]. Therefore, the dorsal hippocampus plays an important role in encoding fear memory, whereas the ventral hippocampus plays a role in fear expression.

Current research indicates that plasticity in the hippocampus (including the CA regions and DG) plays a key role in fear memory along with plasticity in the amygdala, including the lateral amygdala, basolateral nuclear complex, and central nucleus, and in the medial prefrontal cortex (mPFC) [6]. Relative to other brain regions, the hippocampus is highly sensitive to trauma and stressors. Magnetic resonance imaging analyses have often revealed smaller hippocampal volumes in patients with PTSD, particularly in the CA3 and DG regions, and may be considered as a risk factor for vulnerability to PTSD [7,8]. Consistently, animal studies using a single prolonged stress, which is widely used in animal studies on PTSD and fear/anxiety conditions, reported that these conditions lead to dendritic atrophy and loss of dendritic spines in the CA3 region, reduced hippocampal neurogenesis, and mature granule neuronal death in the DG [9,10,11,12,13,14].

Newborn neurons are continuously generated from the division of neural stem cells and neural progenitor cells in restricted regions of the hippocampus of the adult mammalian brain; this process is known as adult hippocampal neurogenesis [15]. Newborn neurons are generated in neurogenic niches, such as the subgranular zone and granule cell layer, within the DG. Newborn dentate granule cells (DGCs) are affected by environmental experience and may participate in hippocampal functions including learning, memory, anxiety, stress regulation, and social behavior. These neurons are generated during the maturation process, which includes the growth of axons and dendritic spines and the formation of synaptic connections. The axons of newborn neurons, known as mossy fibers, are connected to synapses with excitatory pyramidal cells in the CA3 and CA2 regions. Synaptic integration sets a time constraint on the contributions of newborn DGCs to neuronal circuitry in the adult brain [16,17]. Functionally mature newborn neurons are integrated into existing circuits and incorporated into the hippocampal network, which plays a critical role in long-term spatial learning and memory, pattern separation, anxiety, and fear generalization [18,19]. The existing neural circuits, as well as the integration of newborn neurons into the neural circuitry, have been demonstrated to have abnormal connections in fear-related disorders [20,21].

## 5. Hippocampus-Related Neural Circuits in Fear Memory

PTSD is characterized by heightened arousal and resistance to extinction of fear learning and memory [22]. Fear responses, including fear learning, regulation, and extinction, do not occur due to the neural activities of a single brain structure, but rather as a result of the organized activity of multiple brain regions mediated by the synaptic connections between them. Fear conditioning causes the dissociation of brain neural circuits for fear. Several key structures that generate and regulate fear responses to fear conditioning signals have been identified. The hippocampus is primarily used for the encoding and initial storage of contextual fear, whereas the amygdala is essentially used for modulation. Therefore, understanding the neural circuits involved in PTSD is substantial for elucidating its pathogenesis and developing therapeutics to treat it.

### 5.1. Limbic–Frontal Neural Circuits in the Hippocampus

The amygdala, prefrontal cortex, and hippocampus constitute the limbic-frontal neural circuits and are identified as the three key brain regions involved in the learning, regulation, and extinction of fear response in animals [23]. Human studies using functional magnetic resonance imaging have also found that these regions are stimulated by fear conditioning, suggesting the involvement of limbic–frontal neural circuits in this process [24,25]. The neural circuits for fear response contain specific regions, and the amygdala, which is the key region, is located within the medial temporal lobe of the brain. It receives input signals from the thalamus and orchestrates responses to threatening signals by sending outputs to the hypothalamus, basal ganglia, and brainstem to produce defensive behaviors [26]. The amygdala is closely connected to the hippocampus and sends signals to the neurons within it which encode contextual information related to emotional and fear memories from these signals. The hippocampus is involved in the extinction of fear memory and plays a role in the downregulation of the amygdala’s response to these signals [27,28]. The mPFC controls fear response, receives input from the hippocampus, and projects the output onto the amygdala to regulate fear behavior [29]. Contextual expression of fear is influenced by the prelimbic region of the dorsal mPFC, which receives inputs from both the ventral hippocampus and the basolateral amygdala [30]. In contrast, activation of the infralimbic regions of the mPFC is involved in the extinction and inhibition of fear conditioning [31].

Thus, because of the functional interaction between the amygdala and ventral hippocampus, any context associated with a traumatic event can cause fear and anxiety. Maren and Hobin suggested that ventral hippocampal inactivation regulates the contextual modulation of spike firing in the lateral amygdala neurons after fear memory extinction [32], suggesting that the neural projections of the hippocampus and amygdala mediate fear extinction. During fear learning, several neurons that project directly onto the central amygdala are activated, and downstream projections from the central amygdala initiate physiological responses during fear conditioning [33]. The neurons involved in this process are known as fear neurons. In contrast, the stimulus-evoked firing activity of fear neurons are switched off during fear extinction via the activation of extinction neurons, which effectively balances the fear response between the hippocampus and the mPFC.

### 5.2. The Trisynaptic Circuit in the Hippocampus for Fear Memory

The trisynaptic circuit is one of the neural circuits in the hippocampus that involves three major neurons: DGCs, pyramidal neurons in CA3, and pyramidal neurons in CA1. The entorhinal cortex transmits signals from the DG via granule cell fibers, which is known as the perforant pathway. DGCs project onto pyramidal cells in the CA3 region via mossy fibers. CA1 pyramidal neurons receive signals from the Schaffer collaterals of the CA3 pyramidal neuronal axons. Finally, this signal is passed down to the subiculum and into layers IV−VI of the entorhinal cortex. This pathway is necessary for the storage of new and remote memories and the memory consolidation process [34]. Following contextual fear conditioning, blockade of CA3 output via the trisynaptic circuit using CA3-TeTX transgenic mice impaired the acquisition and consolidation of memory when exposed to a novel context of mild footshock [35]. Thus, activation of the trisynaptic circuit by the DG, especially the CA3−CA1 synapse, is important for contextual fear conditioning [36].

### 5.3. Neural Circuitry Basis of Fear Memory

In animal studies, neural circuits related to fear learning, memory, and extinction have been identified using chemogenetic/optogenetic techniques. Chemogenetic/optogenetic techniques provide the ability to modulate neurons and glia in a cell-type, region-specific, and gene-specific manner. Chemogenetics provide the ability to modulate neuronal firing with designer receptors exclusively activated by designer drugs (DREADDs), whereas optogenetics provide precision in controlling neuronal firing with light pulses [37,38,39]. There is strong evidence that neural connections between the hippocampus and amygdala are involved in fear learning, memory, and extinction. Using chemogenetics through the viral vector-mediated expression of the inhibitory muscarinic M4 receptor-based hM4D (Gi)-coupled DREADDs, Ortiz et al. revealed that inactivating the anterior cingulate cortex (ACC) or ventral hippocampus projections to the basolateral amygdala significantly reduced fear generalization to a novel and nonthreatening response [40]. This indicates that the ACC and ventral hippocampus, via projections to the basolateral amygdala, regulate fear generalization. Zhang et al. found that the chemogenetic inhibition of excitatory neuronal activity in the dorsal DG of the hippocampus is directly related to a higher expression of fear memory [17]. In contrast, enhancing neuronal activity in the dorsal DG using DREADDs (hM3D) or optogenetic stimulation reduced the percentage of freezing time, suggesting a reduced expression of fear memory. Using optogenetic techniques, Kheirbek et al. revealed that the dorsal DGCs control memory encoding (not retrieval) of contextual fear conditioning, whereas the ventral DGCs did not affect contextual fear memories but did reduce anxiety [41].

### 5.4. Cellular Factors in the Brain Involved in Fear Memory

The activation of the brain regions involved in each neural circuit following PTSD or fear conditioning can be determined by alterations in the expression of neural activity-related factors. De la Fuente et al. demonstrated that glucose consumption is low in the brain regions known to be associated with memory consolidation, such as the hippocampus and amygdala, indicating low activity of energy-demanding processes, including gene transcription and protein synthesis [42]. Hyperactivation of the amygdala has been associated with fear and hyperarousal in patients with PTSD [43]. During fear conditioning in animal models of PTSD, the expression of *c-Fos* or *c-Jun* (neuronal activity-regulated genes) is increased in several brain regions, including the amygdala, hippocampus, thalamus, and prefrontal cortex [44,45]. Following fear memory extinction, there is a reduction in the activity of fear neurons in the amygdala, and extinction neurons become active [33]. Activation and phosphorylation of extracellular signal-regulated kinase (ERK), cAMP-responsive element binding protein (CREB), and brain-derived neurotrophic factor (BDNF) are key factors in memory formation in the hippocampus and amygdala [46]. Several studies have provided evidence that fear conditioning can induce transient activation of ERK and CREB in these regions [47]. Activation of fear memory resulted in a transient increase in the phosphorylation of ERK and CREB in the amygdala, hippocampus, and mPFC [48]. Chang et al. demonstrated that the expression of BDNF was significantly increased in the amygdala and mPFC, whereas lower BDNF expression was observed in the hippocampus during fear conditioning with footshock [49]. The neural connections were found to be weak within the mPFC, amygdala, and hippocampus during fear conditioning with this method. This evidence supports the idea that, within the amygdala, distinct circuits connect with adjacent brain regions to mediate fear learning, expression, and extinction. Other major cellular factors in the hippocampus that are involved in fear memory and memory formation are the enhanced activities of calcium/calmodulin-dependent protein kinase II (CaMKII), protein kinase A (PKA), and protein kinase C (PKC), which are closely associated with synaptogenesis and long-term potentiation [50]. Inhibition of these factors in the CA1 region using direct inhibitors or antagonists has been shown to reduce learning and fear memory extinction, indicating that these cellular factors are important for fear memory and extinction [51].

Homocysteine is a sulfur amino acid formed by the metabolism of methionine to cysteine. It exerts an excitotoxic action in organotypic cultures from the rat brain cortex and hippocampus [52,53]. In neonatal rats treated with homocysteine, structural and functional changes appear in the brain due to hyperactivity of excitatory neurons [54], resulting in cognitive deficits [55]. Hyperhomocysteinemia has been reported in patients with neuropsychiatric diseases such as Alzheimer’s disease, Parkinson’s disease, schizophrenia, and depression [56]. In addition, hyperhomocysteinemia induces memory deficits via neuronal loss in the hippocampal CA3 region and reduced CREB phosphorylation [57]. According to clinical statistics, serum homocysteine levels are dramatically elevated in patients with PTSD [58,59]. Moreover, the anxious state in a healthy human is positively correlated with homocysteine levels [60]. Put together, these reports suggest that stress or stress-related fear and anxiety may be associated with high levels of serum homocysteine.

## 6. Therapeutic Interventions for Fear-Based Disorders via the Modulation of Proneurogenic Activity and Neural Circuits

We summarized the therapeutic interventions for fear memory and extinction based on the modulation of proneurogenic activity and neural circuits (Table 1). These interventions act as neurogenic enhancers, either by modulating the neurogenic mechanisms discussed in Section 5.4, or by modulating adult hippocampal neurogenesis, ultimately regulating neural circuits related to fear memory. These therapeutic strategies can be used to treat fear-related disorders by improving the function of the brain regions involved in fear memory, particularly the hippocampus, amygdala, and mPFC, or by increasing the expression of cellular factors. In addition, several drugs that act as pro-neurogenic inducers are effective in the prevention of fear learning or extinction of fear memory and related disorders, including PTSD.

D-Cycloserine is a partial agonist of the glycine site of the N-methyl-D-aspartate (NMDA) receptor and has been reported to promote fear extinction in animals and humans [61,62]. In a previous study, the D-cycloserine-treated group exhibited lower levels of fear than the saline-treated control group after the extinction period during fear training. According to this study, D-cycloserine facilitated fear extinction with increased phosphorylated ERK expression in the mPFC and amygdala. Interestingly, the expression of *c-fos* and phosphorylated ERK increased in the mPFC of untrained young rats, indicating that D-cycloserine acts as a proneurogenic inducer [63]. In humans, D-cycloserine administration also attenuates fear extinction and slows reacquisition [64]. However, further studies are required to demonstrate its efficacy in other fear-related disorders and neural circuits.

Sertraline and paroxetine, selective serotonin reuptake inhibitors (SSRIs), are commonly used to treat depression or anxiety-related disorders. These drugs are the first-line treatment for PTSD. When sertraline was administered to prenatal stress-exposed offspring rats, susceptibility to fear stress and traumatic events from mild footshock was reduced [65]. Paroxetine reduces social fear in unfamiliar mice that are exposed to social fear conditioning [66], suggesting the possibility of a novel treatment for fear-related disorders. SSRIs that aim to increase BDNF/tropomyosin receptor kinase B (TrkB) signaling or hippocampal neurogenesis may improve pattern separation and hippocampal function, improve contextual processing, and help modulate fear responses [83]. Fluoxetine, which is also an SSRI used for depression and fear/anxiety-related disorders, exhibited a BDNF/TrkB-dependent effect on the extinction of fear memory in the dorsal and ventral hippocampus, as well as the amygdala and mPFC [67]. Although further studies are required, existing research suggests that SSRIs modulate the activity of brain regions and neural circuits related to fear memory. McAvoy et al. demonstrated that chronic treatment with fluoxetine resulted in increased hippocampal neurogenesis, maturation of newborn dentate granule neurons, and neuronal activity in adult mice, but not in middle-aged mice [68]. Contreras et al. revealed that fluoxetine treatment regulated fear-based chronic stress associated with the microcircuits of limbic–cortical circuits, particularly the lateral septal nucleus and the mPFC [69]. Citalopram administration reduced the overgeneralization of fear memory and extinction of fear learning in auditory cued fear conditioning [70]. Chronic administration of citalopram reduced the expression of the NR2B subunit of the NMDA receptor in the amygdala, which is important for synaptic plasticity and acquisition of fear [71]. Moreover, although not a fear-associated model, there are reports that SSRIs regulate hippocampal synaptic plasticity and structural remodeling by intervening in neural circuits [84,85,86]. This evidence suggests that SSRIs may have therapeutic effects not only on fear memory but also on fear-related disorders, as they influence neural circuit regulation.

Cannabinoids are compounds found in *Cannabis sativa*, also known as cannabis or hemp. The most common cannabinoids are tetrahydrocannabinol (THC) and cannabidiol (CBD). Evidence for the health benefits of CBD suggested that the anti-inflammatory, neuroprotective, and antidepressant effects of CBD may help against psychiatric disorders, such as anxiety and insomnia, and pain [87]. Preclinical studies reported that CBD increased the survival of DGCs in mice and doublecortin-positive neuroblasts, thus promoting hippocampal neurogenesis [88]. CBD administration also increases hippocampal BDNF/ERK/CREB expression, which is important for neuronal survival and maturation [89]. These bodies of evidence suggest that the therapeutic mechanism of CBD possesses proneurogenic efficacy. In fear acquisition and memory, studies have reported that CBD relieved fear and fear-related anxiety by reducing fear learning and memory [70,72]. Clinical studies have reported the potential of THC to enhance neural circuits, particularly ventral mPFC and hippocampus activation, to increase the extinction and recall of fear memory [73,74]. Notably, Rabinak et al. identified the fear-extinction circuitry that involved the ventral mPFC, hippocampus, and amygdala, in a clinical study. These findings suggest that cannabinoids have proneurogenic properties, indicating it has therapeutic potential for fear-based disorders, including anxiety and PTSD.

In addition, dopamine and 5-hydroxytryptamine (5-HT), which show pro-neurogenic properties by increasing hippocampal neurogenesis and modulating neural circuits, also exhibit effective responses to fear-related disorders [90,91]. A recent report suggests that brexpiprazole, a dopamine D2 receptor agonist, is a possible new pharmacological drug against PTSD that promotes the extinction of maladaptive fear memory and modulates the hyperactivation of the amygdala and hippocampus [75]. Moreover, the activation of 5-HT1 receptor in the lateral habenula through an injection of 5-carboxyamidotrypamine maleate salt (5-HT1 receptor agonist) decreased fear acquisition, which was accompanied by decreased AMPA receptor in the hippocampus [76]. Mohammadi-Farani et al. found that the activation of 5-HT3 receptors in the mPFC is an important mechanism of PTSD and fear-related disorders [77]. The 5-HT3 receptor antagonist, ondansetron, increased the fear extinction in an SPS-induced PTSD model, suggesting that blockade of 5-HT3 receptor is a treatment for fear and fear-related disorder.

Neuropeptides act as key modulators of hippocampal neurogenesis and hippocampus-dependent memory via the modulation of the activity of neural stem cells and excitability of DGCs [92]. In particular, neuropeptide Y (NPY) is a polypeptide neurotransmitter that is widely distributed in the brain. The proneurogenic action of NPY on DGCs has been reported both in vitro [93,94] and in vivo [95]. NPY promote the proliferation of neural stem cells through ERK signaling [94]. Previous studies have reported that exogenous NPY administration increased DGC proliferation and neuronal differentiation [95,96]. NPY is considered an endogenous modulator of stress vulnerability and resilience that is selectively released from hippocampal GABAergic interneurons and anteroventral bed nuclei of stria terminalis [78,79,97]. NPY-expressing interneurons of the dorsal DG are activated during fear response, leading to decreased contextual fear memory and increased fear extinction [78]. Neuropeptide S (NPS) also exhibits proneurogenic activity and is involved in modulation of fear memory. Exogenous injection of NPS into the cerebral ventricle or amygdala reduces fear-conditioning activity [80] and increases fear extinction [81]. Interestingly, NPS receptor-deficient mice exhibit generalization of fear memory and anxiogenic phenotypes in a test of anxiety, fear, and stress behaviors, thereby demonstrating the role of NPS in fear memory modulation [82]. Thus, these reports illustrate the important role of neuropeptides in modulating fear memory and extinction.

## 7. Conclusions

**Figure 1:**
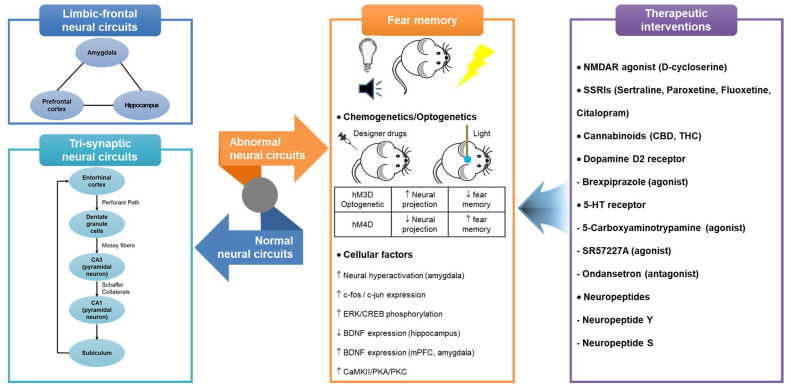
Representation of hippocampus-dependent neural circuits and cellular factors involved in fear memory and therapeutic interventions acting on them. Abbreviations: 5-HT, 5-hydroxytryptamine; BDNF, brain-derived neurotrophic factor; CBD, Cannabidiol; CREB, cAMP response element-binding protein; CaMKII, calcium/calmodulin-dependent protein kinase II; ERK, extracellular signal-regulated kinase; mPFC, medial prefrontal cortex; NMDAR, N-methyl-D-aspartate receptor; PKA, protein kinase A; PKC, protein kinase C; SSRIs, selective serotonin reuptake inhibitors; THC, tetrahydrocannabinol.

In this review, we provide an overview of fear learning and memory associated with PTSD and anxiety-related disorders (Figure 1). Furthermore, the cellular factors and neural circuits involved in fear memory have been summarized, including the neural circuits of the hippocampus and adjacent brain regions studied using novel circuit-based approaches (e.g., chemogenetic and optogenetic techniques). Fear learning and memory depend on the development of long-term potentiation in the hippocampus, amygdala, and mPFC. Neural circuits in these brain regions play key roles in contextual fear, auditory fear, and fear extinction. Previous studies have demonstrated that upregulated responsivity of the amygdala and ACC reduces the responsiveness of the mPFC and the hippocampal function. A recent case study has reported that healthcare workers, veterans, and children may currently be particularly vulnerable to PTSD and stress- or fear-related disorders due to the COVID-19 pandemic [98]. In addition, there have been reports of successful cases of anxiety and stress relief by prescribing therapeutic drugs to such individuals [99,100]. However, the research on neurocircuitry approaches in the relationship between the COVID-19 pandemic and fear-related disorders remains inadequate. Therefore, understanding the neural mechanisms underlying fear memory is essential for the development of novel treatments for psychopathology. Animals and humans differ greatly in the process of fear acquisition and formation of fear memories. Since the process of acquiring fear is largely diverse and the neural circuits involved are very complex, various animal model and human studies should be conducted to characterize the cellular and molecular mechanisms underlying fear-related disorders.

## Figures and Tables

**Table 1:** Summary of therapeutic interventions modulating proneurogenic efficacy and neural circuits for fear-based disorder.

Category	Drug	Model	Treatment	Effects	Reference
NMDAR agonist	D-cycloserine	SD male rat Fear conditioning (light, footshock)	15 mg/kg s.c.	Increased fear extinction	[61]
		SD male rat Fear conditioning (noise, light, footshock)	3.25, 15, 30 mg/kg i.p. 10 μg/side, intra-amygdala infusion	Increased fear extinction	[62]
		SD male rat Fear conditioning (noise, light, footshock)	30 mg/kg i.p.	Increased pERK, c-fos, and iGluR subunits expression in the amygdala and mPFC (PL, IL) during fear extinction period	[63]
		Human (28 participants with acrophobia)	50, 500 mg/day p.o.	Reduction in intense fear of heights within the virtual environment	[64]
SSRIs	Sertraline	Wistar rat Prenatal stress (Immobilization)	5 mg/kg p.o. 3 months	Decreased anxiety-like behaviors and prenatally stressed behaviors Increased fear extinction	[65]
	Paroxetine	CD1 mice Social fear conditioning (unfamiliar mice, footshock)	10 mg/kg Drinking water 14 days	Reduced long-term social fear	[66]
	Fluoxetine	Wistar rat Fear conditioning (noise, light, footshock)	10 mg/kg i.p. 12 days	Reduced fear response via Trk receptor Negative correlation with c-fos Increased BDNF levels in the ventral hippocampus (acute administration)/dorsal hippocampus (chronic administration)	[67]
		Thy1-GFP mice Fear conditioning (light, footshock)	18 mg/kg Drinking water 28 days	Reduced freezing acquisition in contextual fear conditioning Increased hippocampal neurogenesis, newborn neuronal maturation, and neuronal activity in adulthood mice	[68]
		Wistar rat Fear-based chronic mild stress	1 mg/kg i.p. 21 days	Decreased responsivity of lateral septal nucleus projections to the mPFC (PL, IL) regions under stressed conditions	[69]
	Citalopram	C57BL/6 mice Fear conditioning (noise, footshock)	10 mg/kg i.p.	Reduced generalization of fear memory Enhanced extinction of fear memory	[70]
		SD rat Fear conditioning (noise, footshock)	10 mg/kg i.p. 9 days, 22 days	Reduced NR2B expression in the amygdala	[71]
Cannabinoids	Cannabidiol (CBD)	C57BL/6 mice Fear conditioning (noise, footshock)	10 mg/kg i.p.	Reduced generalization of fear memory Enhanced extinction of fear memory	[70]
		Wistar rat Fear conditioning (noise, footshock)	10 mg/kg Infusion	Disrupted memory consolidation by reduction of Arc expression in the dorsal hippocampus	[72]
	Tetrahydrocannabinol (THC)	Human (28 volunteers) Fear conditioning (noise, visual cue)	7.5 mg/day/once p.o.	Increased ventral mPFC and hippocampus activation Decreased fear learning and recall	[73]
		Human (77 volunteers) Fear conditioning (noise, visual cue)	7.5 mg/day/once p.o.	Enhanced fear extinction recall with higher hippocampus activation	[74]
Dopamine D2 receptor	Brexpiprazole (agonist)	C57BL/6 mice Fear conditioning (noise, footshock)	0.3 mg/kg i.p. 7 days	Blocked the maladaptive fear memory Promoted the reversal from PTSD-like fear memory to normal fear memory Normalized the hyperexpression of c-fos in the amygdala and hippocampus	[75]
5-hydroxytryptamine (5-HT) receptor	5-Carboxyamidotrypamine (agonist)	SD rat Fear conditioning (noise, footshock)	1 μg/μL Infusion	Activation of 5-HT1 receptor in the lateral habenula Reduced fear acquisition Decreased long-term potentiation and AMPAR in the hippocampus	[76]
	SR 57227A (agonist) Ondansetron (antagonist)	Wistar rat SPS model	3 μM/0.5 μL 2 μM/0.5 μL	Increased expression of 5-HT3 receptor in the mPFC (IL) Enhanced fear extinction by ondansetron administration	[77]
Neuropeptides	NPY	VGAT-cre mice NPY-GFP mice Fear conditioning (noise, footshock)	Viral vectors (hM3D/hM4D/ preproNPY)	NPY selectively expressed the GABAergic interneurons in the hippocampal DG Enhanced fear extinction Decreased contextual fear memory	[78]
		C57BL/6 mice Fear conditioning (noise, footshock)	NPY_3-36_ (NPY receptor agonist)	Reduced c-fos-expressed cells in the BNSTav Enhanced fear extinction Reduced fear reinstatement	[79]
	NPS	DBA1 mice Fear conditioning (noise, footshock)	0.01, 0.1, or 1 nmol NPS/side Intra-amygdala infusion	Inhibited expression of fear response	[80]
		C57BL/6 mice Fear conditioning (noise, footshock)	10 μM/0.5 μL Intra-amygdala infusion	Increased extinction of fear memory Restored excitatory synaptic activity in lateral amygdala projection neurons	[81]
		NPS receptor-deficient mice Fear conditioning (noise, footshock)	1 nmol NPS/side Infusion into the lateral ventricle	Inhibited spontaneous locomotor activity No effect on fear learning and adaptation	[82]

Abbreviations: 5-HT, 5-hydroxytryptamine; 5-HT1, 5-hydroxytryptamine (serotonin) receptor-1; 5-HT3, 5-hydroxytryptamine (serotonin) receptor-3; BDNF, brain-derived neurotrophic factor; BNSTav, anteroventral bed nuclei of stria terminalis; CBD, Cannabidiol; DG, dentate gyrus; GABA, γ-aminobutyric acid; pERK, phosphorylated extracellular signal-regulated kinase; GFP, green fluorescent protein; GluR, ionotropic glutamate receptor; IL, infralimbic; i.p., intraperitoneal; mPFC, medial prefrontal cortex; NMDAR, N-methyl-D-aspartate receptor; NPS, neuropeptide S; NPY, neuropeptide Y; NR2B, N-methyl-D-aspartate receptors 2B subunit; PL, prelimbic; p.o., per oral; PTSD, posttraumatic stress disorder; s.c., subcutaneous; SD rat, Sprague–Dawley rat; SPS, single prolonged stress; SSRI, selective serotonin reuptake inhibitor; THC, tetrahydrocannabinol; TrkB, tropomyosin receptor kinase B.

## Data Availability

The datasets supporting the conclusions in this study are contained in the article.
